# The impact on early diagnosis and survival outcome of M-protein screening-driven diagnostic approach to multiple myeloma in China: a cohort study

**DOI:** 10.7150/jca.32103

**Published:** 2019-08-27

**Authors:** Jing Li, Yue Wang, Peng Liu

**Affiliations:** Department of Hematology, Zhongshan Hospital, Fudan University, Shanghai, China

**Keywords:** Multiple myeloma, monoclonal gammopathy, cancer screening, China

## Abstract

**Background**: Most multiple myeloma (MM) patients in China were diagnosed only until severe complications occurred. The incidence of MM in China is 7.3 times lower than that of the United States, which could have been underestimated due to high rate of miss diagnosis and diagnostic delay in China. In Zhongshan Hospital Fudan University, serum protein electrophoresis (SPEP) is routinely incorporated into liver function test panel, and therefore providing us unique opportunities to carry out a hospital-population-based M-protein screening by SPEP and establish a screening-driven diagnostic approach.

**Methods**: A retrospective cohort study analyzing data of patients screened by SPEP and diagnosed with MM from 2014 to 2017 was performed. We compared the baseline features and outcome of patients diagnosed via screen-driven approach and symptom-driven approach. We also analyzed the efficacy of M-protein screening.

**Results**: A total of 690,000 people were screened and 335 eligible MM patients were identified, among which 151 of them were diagnosed via screening-driven approach. Compared to symptom-driven group, patients in screening-driven group had earlier ISS stage disease (*P* = 0.025), lower frequency of anemia (*P* = 0.000) and bone lesion (*P* = 0.012), and lesser number of end-stage symptoms (*P* = 0.000). M-protein screening approach demonstrated significantly (*P* = 0.029, HR: 0.415) better outcome (3-yr OS, 76.9%) than those in symptom-driven subgroup (3-yr OS, 46.6%) after being adjusted for age, gender, CRAB symptoms and ECOG score with a Cox proportional hazards model. Furthermore, the annual incidence of MM in Zhongshan Hospital screening population is 20.82/100,000, much higher than that in the whole China despite of selection bias.

**Conclusion**: We concluded that the actual MM incidence in China may have been underestimated and M-protein screening in hospital population by SPEP is an effective approach to improve early diagnosis rate and outcome.

## Background

Multiple myeloma (MM) is a plasmacytic malignancy preceded by its preliminary state, monoclonal gammopathy of undetermined significance (MGUS) 1, 2, and often results in end-organ symptoms including hypercalcemia, renal insufficiency, anemia and osteolytic bone lesion (CRAB).The annual incidence of MM in China is 0.9/100,000, much lower than that in the United States (6.56/100,000)^3^, ^4^. Despite racial disparities and hereditary distinction, the difference in incidence of MM between China and the US may also lie in high miss diagnosis rate and high percentage of diagnostic delay in China. Most myeloma patients in China were diagnosed and treated only until severe complications occurred, with 85.8% of patients staged as Durie-Salmon stage III at diagnosis 5. Meanwhile, Chinese myeloma patients suffer from an inferior outcome, with a 5-year overall survival (OS) rate lesser than 50% in patients treated at three famous myeloma centers in China 5, lower than that of MM patients in the USA (5-year OS, 50.4%) 4. However, according to a SEER (Surveillance, Epidemiology and End Result Program) analysis studying MM patients in the USA, the Asian population had the best outcome compared with other races 6, which is contradictory to what was observed in China. Besides difference in treatment, we hypothesized high miss diagnosis rate and diagnostic delay could also explain the difference in outcome.

In an effort to increase the diagnostic rate and improve the outcome for myeloma patients in China, we established a diagnostic approach driven by monoclonal immunoglobulins (M-protein) screening in our institute, Zhongshan Hospital Fudan University. In Zhongshan Hospital, a large national reference center in Shanghai delivering medical service for over 4,000,000 patients from all over the country annually, serum protein electrophoresis (SPEP) is routinely incorporated into liver biochemical and function tests. Hence, every patient having liver biochemical and function tests were indeed screened for M-protein by SPEP simultaneously in our institute. With routine SPEP screening, we had the unique opportunities to observe the prevalence of monoclonal gammopathy in a large-scale hospital population and to identify patients with early-stage myeloma via the M-protein screening-driven diagnostic approach.

Here, we retrospectively evaluated the impact of the M-protein screening-driven diagnostic approach on baseline characteristics and outcome of patients with MM. Furthermore, we assessed whether the morbidity of multiple myeloma in China was really as low as reported, and whether diagnostic pattern was one of the main cause of later staging and poorer prognosis. As a secondary aim, we studied the efficacy of M-protein screening in hospital population.

## Methods and Patients

### M-protein screening-driven diagnostic approach

**Figure 1:**
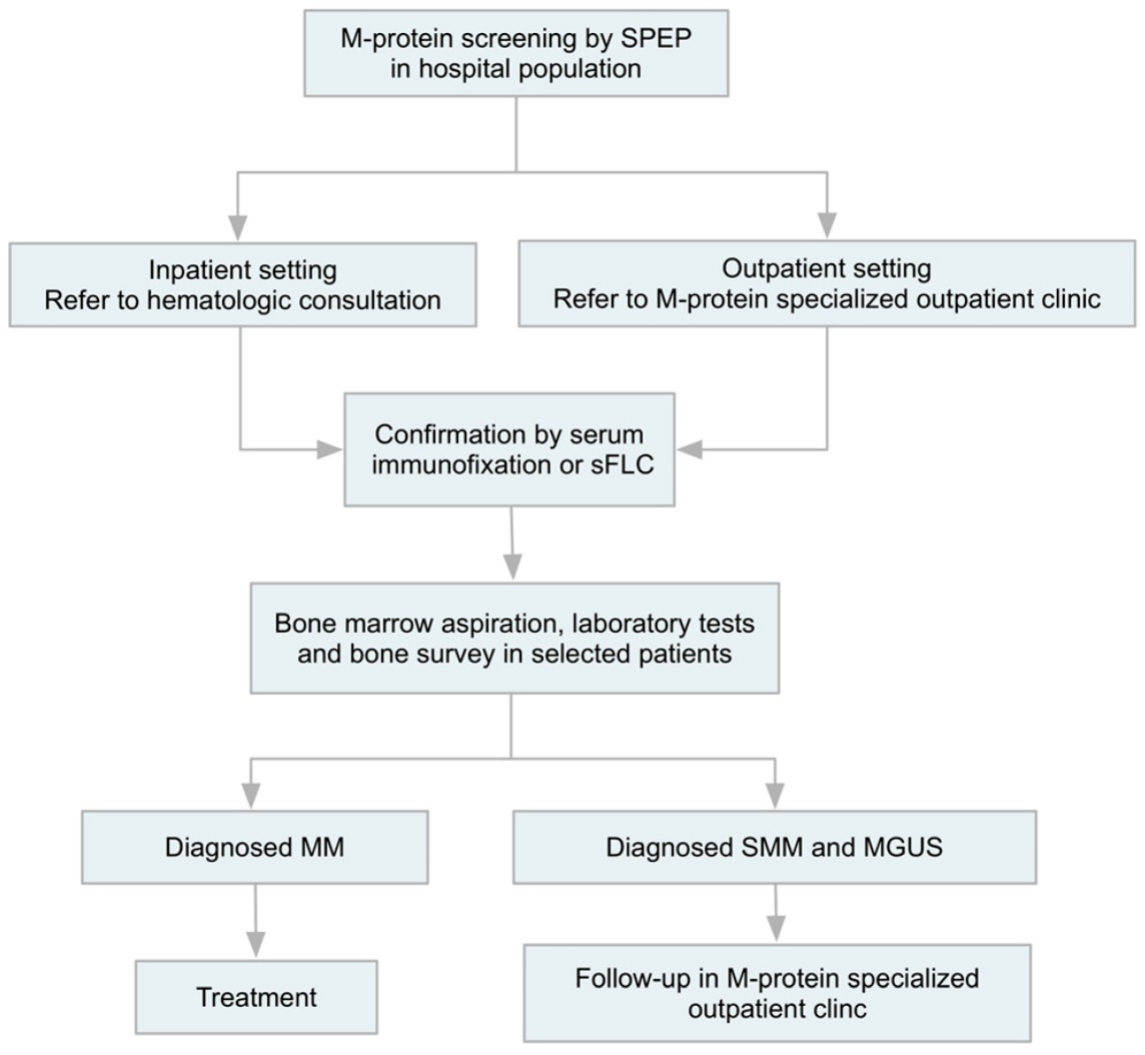
M-protein screening-driven diagnostic approach. Patients with abnormal narrow band in SPEP would be referred to hematology consultation (inpatient setting) or specialized M-protein outpatient clinic (outpatient setting) for further investigation, including confirmation of monoclonal gammopathy by immunofixation electrophoresis and/or serum free light chain assay, followed by bone marrow test, laboratory tests and bone survey in selected patients. Patients diagnosed with MM would receive treatment and those with SMM or MGUS would be carefully followed-up in specialized clinic. SPEP, serum protein electrophoresis; sFLC, serum free light chain; MM, multiple myeloma; SMM, smoldering myeloma; MGUS, monoclonal gammopathy of undetermined significance.

We initiated a standardized M-protein screening procedure based on SPEP screening in hospital population (**Figure 1**), given every patient receiving liver biochemical tests were screened for M-protein by SPEP in our institute. Patients detected with an abnormal narrow band in SPEP would be referred to hematology consultation (inpatient setting) or specialized M-protein outpatient clinic (outpatient setting) for further investigation, including confirmation of monoclonal gammopathy by immunofixation electrophoresis and/or serum free light chain assay. For patients with accompanied symptoms of anemia, renal insufficiency, bone pain, concurrent infection and fatigue, those who had high risk features (including serum monoclonal protein >15 g/l, IgA or IgM protein type, or an abnormal free light chain ratio) 7, and patients who were willing to undergo thorough check-up, further examination including bone marrow aspiration and biopsy, bone survey and relevant laboratory tests would be carried out. Patients diagnosed with MM would receive treatment and those with SMM or MGUS would be carefully followed-up in specialized clinic.

### Patients

In this retrospective cohort study, we examined records of hospitalized newly diagnosed MM patients with complete data from January 2014 to December 2017. MM Patients with concurrent cardiac amyloidosis were excluded due to distinct prognosis. Patients were categorized by the means of detection of the disease. Patients were considered in screening-driven group if they had an incidental finding of M-protein during workup of unrelated medical conditions or routine body check-up and were diagnosed via the M-protein screening-driven diagnostic approach. Symptom-driven group included patients visited or were referred to hematologic department due to suspected myeloma-related end-organ damage.

Institutional ethics committee approved the study and informed consent was waived.

### Statistical Analysis

Baseline characteristics, treatment, overall survival (OS) and progression-free survival (PFS) were compared. PFS was calculated from the date of diagnosis to the date of death or first progression/relapse. OS was defined as the time from the date of diagnosis to the date of last follow-up or death. Patients without events or death at the time of last follow-up were recorded as censored data. Statistical computations were performed using Stata (version 12.0; StataCorp LP, College Station, TX, USA) and SPSS (version 19; IBM, New York, USA) software. All statistical tests were two-tailed, and statistical significance was set at *P* < 0.05. Student's t-test was performed to determine a statistically significant association of diagnostic pattern and patients' baseline characteristics in our cohort. Survival curves were constructed using the Kaplan-Meier curves and log-rank tests to assess the differences between the groups. Adjusted odds ratio (OR) with 95% confidence intervals (95% CIs) were calculated using Cox proportional hazards models. Univariate and multivariate Cox proportional hazards of diagnostic pattern and OS for patients with MM were analyzed.

## Results

### Screening and Diagnosis

This study included patients who received liver biochemical tests, who were routinely screened for M-protein by SPEP between January 2014 and December 2017 in Zhongshan hospital Fudan University. During this period, a total of 690,000 people were screened by SPEP and 335 previously untreated MM patients were diagnosed (patients with concurrent cardiac amyloidosis excluded). Among the 335 eligible MM patients, 151 of whom were diagnosed through M-protein screening-driven approach, 184 were diagnosed through symptom-driven diagnostic pattern.

### Characteristics of the Patients at Baseline

The median age of these patients was 64 (range: 34 - 87) years. After pathological diagnosis, 242 (72.24%) patients received a Bortezomib-based regimen for initial treatment.** Table 1** summarizes the baseline features of these 335 patients. Compared to symptom-driven group, patients in screening-driven group had earlier ISS stage disease (*P* = 0.025), lower frequency of anemia (*P* = 0.000) and bone lesion (*P* = 0.012), and lesser number end-stage symptoms (*P* = 0.000). The DS stage of the two groups was also close to a statistical difference (*P* = 0.054). In terms of age, gender, and therapy, there was no significant intergroup difference.

To further assess the correlation between diagnostic pattern and disease staging, Spearman correlation analysis was performed and diagnostic pattern was found to be correlated with ISS stage (*P* =0.021) and DS stage (*P* = 0.021). Hence, we suggest that M-protein screening contributed to detect patients at an earlier stage and reduce diagnostic delay.

### Survival

**Figure 2:**
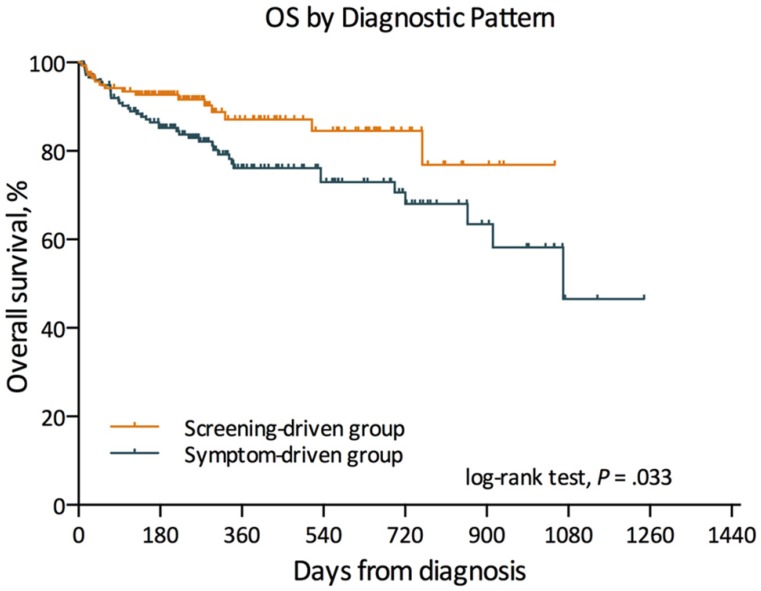
Kaplan-Meier estimates of overall survival in different subgroup.

Kaplan-Meier survival analysis was performed to assess the associations between diagnostic mode and prognosis of MM patients. The result revealed that patients diagnosed via M-protein screening demonstrated significantly (*P* = 0.033) better outcome (3-yr OS, 76.9%) than those of symptom-driven subgroup (3-yr OS, 46.6%) (**Figure 2**). The median follow-up length was 9 months (range: 1 - 41 months).

In univariate Cox regression analysis, diagnostic pattern showed statistically significant effect on OS (*P* = 0.036, HR: 0.537, 95% CI: 0.301 - 0.959). In addition, clinical features including ISS stage (*P* = 0.024, HR: 1.147, 95% CI: 1.052 - 2.057), ECOG score (*P* = 0.007, HR: 1.238, 95% CI: 1.238 - 3.814), initial treatment with Bortezomib-based regimen (*P* = 0.014, HR: 0.505, 95% CI: 0.293 - 0.872), renal insufficiency (*P*=0.004, HR: 2.702, 95% CI: 1.379-5.292) and hypercalcemia (*P*=0.021, HR: 2.530, 95% CI: 1.147-5.580) were also related to OS of MM patients (**Table 2**).

In further multivariate COX analysis which involved gender, age, ISS stage, hypercalcemia, renal insufficiency, anemia, bone lesion, ECOG score and diagnostic pattern, patients diagnosed via M-protein screening demonstrated significantly better outcome than those in symptom-driven subgroup (*P* = 0.029, HR: 0.415, 95% CI, 0.187 - 0.915). ISS stage (*P* = 0.037, HR: 1.870, 95% CI: 1.037 - 3.984), bone lesion (*P* = 0.018, HR: 2.732, 95% CI: 1.192-6.270) and ECOG score > 2 (*P* = 0.023, HR: 2.388, 95% CI: 1.127 - 5.060) were also independent adverse prognostic factors of OS (**Table 3**).

### Efficacy analysis

In this study, the sensitivity of SPEP was 81.79%, the specificity was 99.90%, the positive predictive value was 28.66%, and the negative predictive value was 99.99%.

In addition, although M-protein screening in hospital populations exist a large selection bias, the annual incidence of MM in Zhongshan hospital M protein screen population is 20.82/100,000, which is much higher than that reported in whole China (0.9/100,000)^3^.

The M-protein screening-driven approach was initiated in the end of 2014. Since then, institutional myeloma diagnostic rate has consistently increased. From 2014 to 2017, more and more patients were diagnosed through screening, and more and more of our newly diagnosed myeloma patients were diagnosed each year (7/34 (20.59%) in 2014, 25/50 (50.00%) in 2015, 32/73 (43.83%) in 2016, 87/178 (48.88%) in 2017). In addition, we further compared the disease stage of patients diagnosed in different year via analysis of variance (ANOVA) and found that the ISS stage (*P* = 0.006) and DS stage (*P* = 0.000) were statistically different in patients diagnosed in different year, and the proportion of early stage MM increased year by year.

## Discussion

MM is a hematological neoplasm which characterized by heterogeneous genetic abnormalities and an extensive range of clinical outcomes. According to previous studies 8-10, there exists significant difference in incidence, baseline characteristics and outcome between patients with MM in China and those in the USA:

1) The annual incidence of MM in China is 7.3 times lower than that in the United States.

2) More Chinese patients were found to have advanced-stage disease at diagnosis than the USA patients: DS III (85.8% vs 66%) and ISS III (48.3% vs 39%).

3) Chinese myeloma patients suffer from an inferior outcome: 5-year OS (China: lesser than 50% vs the USA: 50.4%) 4, 5.

Although environment and race could contribute to this difference 6, 11, 12, we suggest that medical factors may also play critical roles in the difference. As we reported before, many Chinese people do not receive routine body examination and often delay seeking health service. Furthermore, as an uncommon disease, multiple myeloma is sometimes not well-recognized by primary care physicians in China. Therefore, most myeloma patients in China were diagnosed at an advanced-stage, usually accompanied by obvious symptoms and severe complications including renal failure, pathological bone fracture, anemia and hypercalcemia. Only a few patients were diagnosed at early stage by incidental finding of monoclonal gammopathy.

Cancer screening has been proved to be a cost-efficient solution to increase early cancer detection and improve survival in several types of malignancy, including lung cancer, breast cancer and colorectal cancer 13-15. Although M-protein screening for detection of early myeloma in normal population is not currently recommended 16, the impact of myeloma screening in hospital population has not been studied. As an inexpensive and easy screening procedure to detect M protein 7, 16, SPEP is an essential component of liver biochemical and function test in our institute. Thus, M protein-screening has been indeliberately implemented in our hospital on every patient receiving liver function test. At the end of 2014, we initiated a standardized M-protein screening-driven diagnostic procedure to deal with the patients with abnormal findings in SPEP screening. In this retrospective study, we analyzed 690 thousand people received SPEP in our hospital, 335 newly diagnosed MM cases during the past four years since the screening procedure carried out. Our study confirmed that patients diagnosed via M-protein screening demonstrated earlier ISS stage disease, lower frequency of anemia, bone lesion and lesser number CRAB symptoms than those in symptom-driven subgroup. Patients in screening-driven diagnosed group had prolonged OS. Early detection and prolonged survival resulted from screening implies improvement in health-related Quality of Life and more opportunities to receive further lines of treatment when disease relapses, which is critical for Chinese MM patients, given that most of them were diagnosed and treated only after severe complications developed.

In this study, we further investigated the efficacy of myeloma screening in hospital population. The high sensitivity of SPEP enables it to meet the requirement for MM screening in a specific population 17, 18. In addition, SPEP can be incorporated into routine liver function examination and its cost is inexpensive. These features also meet the demands of health economics for screening test 19-21. Although the study was carried out in a specific hospital population which had a big selection bias compared to community population 22. But for a relatively low incidence rate disease, screening in certain high-risk groups also satisfied the requirement of health economics, and the results also showed that in hospital population, the incidence rate of MM is much higher. Hence, we proposed M-protein screening has potentially significant economic benefits and could be applied in hospital population.

In addition, the results of M-protein screening in the hospital population showed that the incidence of multiple myeloma in China was significantly higher than reported before (20.82/100,000 vs 0.9/100,000) 3. Moreover, during the study, the proportion of patients diagnosed by M-protein screening increased year by year, and most of the patients diagnosed through M-protein screening would have suffered from delayed diagnosis or miss diagnosis before the screening-driven diagnostic approach was carried out. Our data imply that the exact incidence of MM in Chinese population might have been underestimated.

This was the first report reveals increased detection of early stage myeloma and remarkable survival improvement resulted by M-protein screening in hospital population in China. However, our study has several limitations. First, this study was a retrospective study and all the patients in our study were from a single center. Second, even though the number of patients in our study was relatively large, we only evaluated the value of M-protein screening in hospital population which has a relatively large selection bias compared with community population. However, the main purpose of our study is to improve the early diagnosis rate of multiple myeloma patients, and to predict through a relatively large number of random people whether the incidence rate of patients with multiple myeloma in China was seriously underestimated. Third, the follow-up time of our study was relatively short, the median expectancy life of screening subgroup patients was unknown, further follow-up needs to be updated. To solve this problem, a long follow-up time multicenter-clinical prospective trial through general investigation could be conducted in the future.

## Conclusions

We carried out a hospital-population M-protein screening by SPEP and established an M-protein screening-driven diagnostic approach. We confirmed that M-protein screening is an effective method which can improve the early diagnosis rate of multiple myeloma patients, prolong their overall survival time. And the morbidity of multiple myeloma in China maybe not as low as reported. The low morbidity was not only because of ethnic disparities and hereditary distinction, but also high rate of miss diagnosis and diagnostic delay.

## Figures and Tables

**Table 1:** Characteristics of 335 MM patients

Clinical Features	Screening-driven (n=151)	Symptom-driven (n=184)	*P*-value
Age			
Median (range), year	64(37-87)	64(34-85)	0.194
Male sex, No (%)	93(61.58)	116(63.04)	0.785
DS stage, No (%)			0.054
I	21(27.15)	20(10.87)	
II	28(18.54)	18(9.78)	
III	102(67.55)	146(79.35)	
ISS stage, No (%)			0.025
I	59(39.07)	48(26.09)	
II	40(26.49)	54(29.35)	
III	52(33.11)	82(44.56)	
CRAB symptom, No (%)			
Hypercalcemia	10(6.62)	16(8.69)	0.482
Renal Insufficiency	43(28.48)	55(29.89)	0.778
Anemia	56(37.09)	130(70.65)	0.000
Bone Lesion	83(54.97)	170(92.39)	0.012
Bortezomib, No (%)	111(73.51)	131(71.19)	0.309
ECOG, No (%)			0.786
<2	116(76.82)	139(75.54)	
≥2	35(23.18)	45(24.46)	

^a^ CRAB referred to four major end-organ damages of MM, including hypercalcemia, renal insufficiency, anemia and bone lesion. ^b^ Renal insufficiency was defined as serum creatinine at diagnosis > normal upper limit. ^c^ Anemia was defined as hemoglobin < 120g/L at diagnosis. ISS, International Staging System; DS, Durie-Salmon Staging System.

**Table 2:** Univariate Cox Proportional Hazards Analysis of factors and Overall Survival for Patients

Variables	HR	(95% CI)	*P*
Diagnostic Pattern	0.537	0.301 - 0.959	0.036
Gender	0.668	0.379 - 1.177	0.163
Age	1.012	0.987 - 1.039	0.326
DS stage	0.983	0.649 - 1.488	0.936
ISS stage	1.147	1.052 - 2.057	0.024
Hypercalcemia	1.288	0.751 - 2.210	0.357
Renal Insufficiency	1.718	1.020 - 2.892	0.042
Anemia	2.099	1.028 - 4.290	0.042
Bone Lesion	2.392	1.742 - 5.025	0.021
Bortezomib-based induction therapy	0.505	0.293 - 0.872	0.014
ECOG score > 2	2.173	1.238 - 3.814	0.007

ISS, International Staging System; DS, Durie-Salmon Staging System; ECOG, performing status score of Eastern Cooperative Oncology Group.

**Table 3:** Multivariate Cox Proportional Hazards Analysis of factors and Overall Survival for Patients

Variables	HR	(95% CI)	P
Diagnostic Pattern	0.415	0.187 - 0.915	0.029
Gender	0.532	0.268 - 1.396	0.243
Age	1.018	0.983 - 1.055	0.296
ISS stage	1.870	1.037 - 3.371	0.037
Hypercalcemia	1.679	0.706 - 3.984	0.241
Renal Insufficiency	1.275	0.558 - 2.913	0.565
Anemia	1.616	0.600 - 4.350	0.342
Bone Lesion	2.732	1.192 - 6.270	0.018
ECOG score > 2	2.388	1.127 - 5.060	0.023

ISS, International Staging System; DS, Durie-Salmon Staging System; ECOG, performing status score of Eastern Cooperative Oncology Group.

## Abbreviations

CRAB: hypercalcemia, renal insufficiency, anemia, bone lesion
DS: Durie-Salmon Staging System
ECOG: Eastern Cooperative Oncology Group
HR: hazard ratio
ISS: International Staging System
M-protein: monoclonal immunoglobulin
MGUS: monoclonal gammopathy of undetermined significance
MM: multiple myeloma
OS: overall survival
PFS: progression free survival
SEER: Surveillance, Epidemiology, and End Results Program
SPEP: serum plasma electrophoresis
